# Differential Diagnosis of Urticarial Lesions

**DOI:** 10.3389/falgy.2022.808543

**Published:** 2022-06-16

**Authors:** Ana Luísa Matos, Carolina Figueiredo, Margarida Gonçalo

**Affiliations:** ^1^Dermatology Department, Coimbra University Hospital Center, Coimbra, Portugal; ^2^Faculty of Medicine, Dermatology Department, University of Coimbra, Coimbra, Portugal

**Keywords:** urticaria, angioedema, urticarial syndromes, autoinflammatory syndromes, chronic spontaneous urticaria

## Abstract

Urticaria is a mast cell-dependent disease, characterized by the presence of wheals, angioedema, or both in the absence of systemic symptoms. It is a common disease worldwide, with an important health burden especially in chronic situations, that last more than 6 weeks. Although urticaria is usually a straightforward diagnosis, some diseases presenting with urticarial lesions must be excluded, particularly urticarial vasculitis and auto-inflammatory syndromes. In these settings additional atypical features are often present (long-lasting lesions, bruising, fever, malaise, arthralgia), allowing the clinician to suspect a diagnosis other than urticaria. The authors propose an approach based on these atypical features, the presence or absence of systemic symptoms and on skin histopathology as well as some blood parameters.

## Introduction

Urticaria is characterized by mast cell-dependent wheals, angioedema, or both in the absence of systemic symptoms. Urticaria can be acute or chronic (recurrent signs and symptoms for more than 6 weeks), in the latter case spontaneous and/or inducible (1). Chronic spontaneous urticaria (CSU), the most common form of chronic urticaria (CU), presents with transient wheals, angioedema or both, without any definite triggers. Wheals are pruritic, pink or pale swellings of the superficial dermis that, by definition, resolve in <24 h. Wheals may be round or polycyclic and have various sizes, or may be pale, eventually with an “orange peel” appearance, surrounded by erythema and can affect any area of the body usually in an asymmetric distribution. Angioedema is characterized by swellings that involve the deeper dermis and the subcutaneous or submucosal tissue. Lesions tend to have less precise limits, usually have a normal skin color and are more frequently painful than pruritic. Angioedema can last longer than 24 h but resolves completely over a few days.

Acute urticaria is a common, usually self-limited entity. Although mainly idiopathic, the most commonly identified causes of acute urticaria are infections, followed by drugs, food and hymenoptera venom allergy (2). Food constituents can behave either as allergens (proteinic molecules as tropomyosin from seafood, ovalbumin from egg) or pseudoallergens (non-proteinic molecules like salicylates, benzoic acid). Physical activity can also induce acute urticaria as in exercise-induced urticaria. Oral allergy syndrome represents a mucosal allergic contact urticaria in people sensitized to common pollens, due to IgE cross-reactivity between homologous pollen allergens and various plant foods. It is the most prevalent food allergy, and, even though symptoms are usually mild, self-limiting and localized to the oropharyngeal mucosa, they may sometimes become generalized and life-threatening, with cutaneous manifestations including urticaria (3).

Chronic urticaria has a prevalence of 0.5%−3% and typically persists for months to years. CSU has no obvious cause, but autoimmunity or autoallergy plays an important role in most cases and external triggers, like drugs, infections or stress, can exacerbate it. Inducible urticaria includes a heterogeneous group of conditions elicited mainly by physical stimuli (cold, heat, light, pressure, etc.) or by exercise (cholinergic urticaria). Patients usually identify the trigger although it is important for the physician to confirm it and establish thresholds of reactivity (4). Inducible urticaria can also present with concomitant systemic manifestations, that can occasionally be life-threatening, namely in cold-induced or cholinergic urticaria (5).

## Differential Diagnosis Of Urticaria

The diagnosis of urticaria is usually straightforward, but several mimickers need to be considered in case of an atypical clinical history or physical examination (6) (Table 1). The distinction between wheals and urticarial lesions can be useful in determining when to suspect another diagnosis. Atypical urticarial lesions can be infiltrated and long-lasting (>24 h), coexist with other elementary skin lesions (papules, vesicles, hemorrhages), resolve with hypo/hyperpigmentation or scaling, may have a more symmetric distribution and angioedema is usually absent (4). The presence of systemic symptoms (fever, malaise, arthralgia) is also unusual and should discourage a diagnosis of urticaria. There are several systemic disorders that can present with urticarial lesions, including urticarial vasculitis, connective tissue diseases, hematologic diseases and autoinflammatory syndromes. All these conditions may be considered as differential diagnosis of urticaria (7). Angioedema is associated with CSU in more than 50% of the cases (8), often aggravating the disease burden (9), but when it occurs alone and particularly with associated systemic symptoms, the hypothesis of a bradykinin-mediated angioedema needs to be considered (10).

**Table 1:** Clinical entities with acute and chronic urticarial lesions.

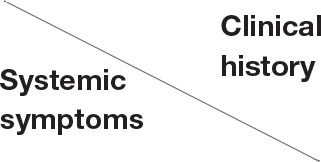	**Acute**	**Chronic**
Present	Anaphylaxis Maculopapular drug exanthem Viral exanthem Erythema multiforme Sweet's syndrome	HUV/HUVS Hypereosinophilic syndromes Cryopyrin-associated periodic syndromes Schnitzler syndrome Adult-onset Still disease Gleich syndrome
Absent	PLE Maculopapular cutaneous mastocytosis Bullous pemphigoid EAC Autoimmune progesterone dermatitis Urticarial dermatitis NUV	

*EAC, Erythema annulare centrifugum; HUV, hypocomplementemic urticarial vasculitis; HUVS, hypocomplementemic urticarial vasculitis syndrome; NUV, normocomplementemic urticarial vasculitis; PLE, polymorphic light eruption*.

When first evaluating a patient with a presumable diagnosis of urticaria, the acute and chronic subtypes may not be discernable. In both settings, other diagnosis may have to be considered, therefore, an approach based on the particular aspects of the lesions and presence or absence of accompanying systemic symptoms and the number of previous episodes seems to be the best clinical strategy.

### Differential Diagnosis in Acute Urticaria

**Figure 1:**
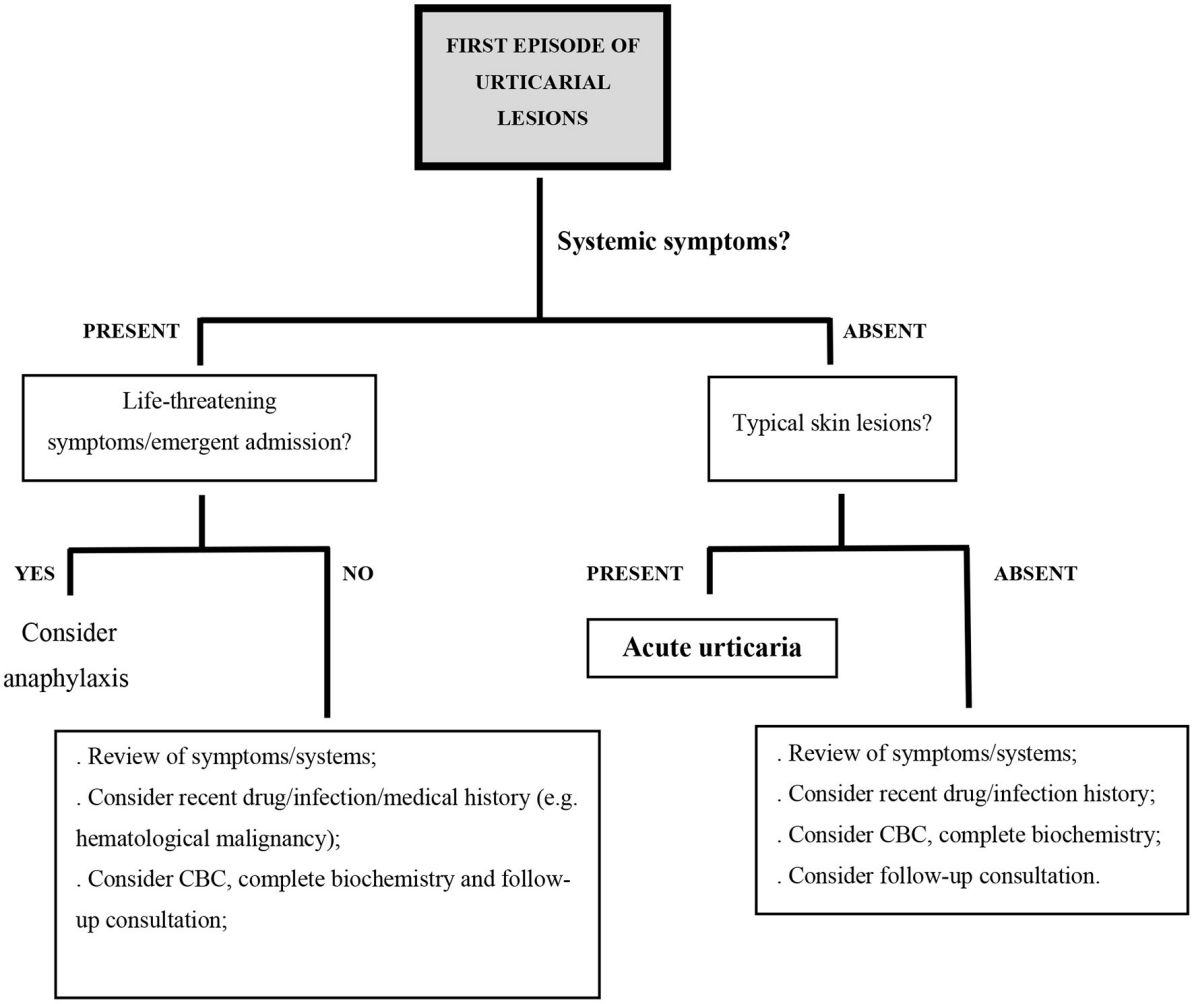
Proposed diagnostic algorithm in the setting of acute urticarial lesions; CBC, complete blood count.

A first episode of urticarial lesions without any accompanying symptoms is not always acute urticaria. When some of the previously mentioned atypical characteristics are present, other diagnosis should be considered (Figure 1).

#### Polymorphic Light Eruption

Polymorphic light eruption usually occurs in spring and consists of symmetrically distributed itchy, polymorphic, erythematous skin lesions that appear after sun exposure and persist for several days (11).

#### Maculopapular Cutaneous Mastocytosis

Maculopapular cutaneous mastocytosis is characterized by multiple hyperpigmented macular or maculopapular lesions that urticate within a few minutes when rubbed (12).

#### Bullous Pemphigoid

Bullous pemphigoid usually begins with a non-specific pruritic rash, occasionally with an urticarial appearance, but lesions tend to persist for days and may progress to bullae. It may be similar to some urticarial dermatoses in pregnancy, e.g. *pemphigoid gestationis*.

#### Erythema Annulare Centrifugum

Erythema annulare centrifugum is characterized by solitary or multiple erythematous, ring-shaped and polycyclic plaques that slowly spread peripherally and may show a characteristic slight scaling behind the advancing edge.

#### Autoimmune Progesterone Dermatitis

Autoimmune progesterone dermatitis is triggered by hypersensitivity to progesterone. Variable skin lesions, resembling wheals or eczema, aggravate cyclically in the premenstrual period (11).

#### Urticarial Dermatitis

Urticarial dermatitis occurs mostly in elderly patients and presents with highly pruritic eczematous and urticarial lesions, simultaneously or sequentially. It is difficult to treat and may be idiopathic or represent the initial presentation of several skin diseases, namely bullous pemphigoid or drug eruptions (13–15). All these clinical entities can also present as recurrent dermatosis and participate in the differential diagnosis of both acute and chronic urticarial lesions.

Otherwise, in the setting of acute urticarial lesions accompanied by systemic symptoms, the clinician should always consider some differential diagnosis. Anaphylaxis with acute urticaria occurs after exposure to an allergen, such as food, medications or insect venom, which trigger the release of vasoactive mediators from mast cells and basophils, often *via* an IgE-mediated pathway. Anaphylaxis is likely when there is an acute onset of generalized wheals and/or angioedema accompanied by respiratory symptoms, reduced blood pressure, syncope, gastrointestinal symptoms, incontinence or uterine cramps (16). Acute urticaria present for hours or days is not likely to evolve into anaphylaxis.

#### Maculopapular Drug Exanthem

Maculopapular drug exanthem is a T-cell mediated reaction that can occur within a few days to 3 weeks of the onset of almost any drug. There is usually a symmetrical eruption of confluent red macules and urticarial papules that begin on the upper trunk and progress distally, persist for several days and evolve into desquamation, sometimes accompanied by systemic symptoms. Viral exanthem may also present as a macular, maculopapular, urticarial, or vesicular reaction that lasts a few days and may be associated with mucosal lesions, fever or other systemic symptoms. Erythema multiforme is an acute eruption of dull red, macular, papular or urticarial lesions with a target appearance. Lesions are preferentially distributed on distal extremities and tend to appear in successive crops for a few days, slowly enlarge, and fade in 1–2 weeks. Erythema multiforme major is usually accompanied by mucosal erosions and systemic symptoms such as fever. On the other hand, urticaria multiforme, an entity sometimes difficult to distinguish from erythema multiforme, is a benign cutaneous hypersensitivity response seen in pediatric patients characterized by the acute and transient onset of urticarial lesions with a dusky quality.

#### Sweet's Syndrome

Sweet's syndrome (acute febrile neutrophilic dermatosis) is characterized by fever and acute onset of painful, erythematous papules, plaques or nodules, often with a pseudovesicular aspect, that persist for days to weeks.

### Differential Diagnosis in Chronic Urticaria

**Figure 2:**
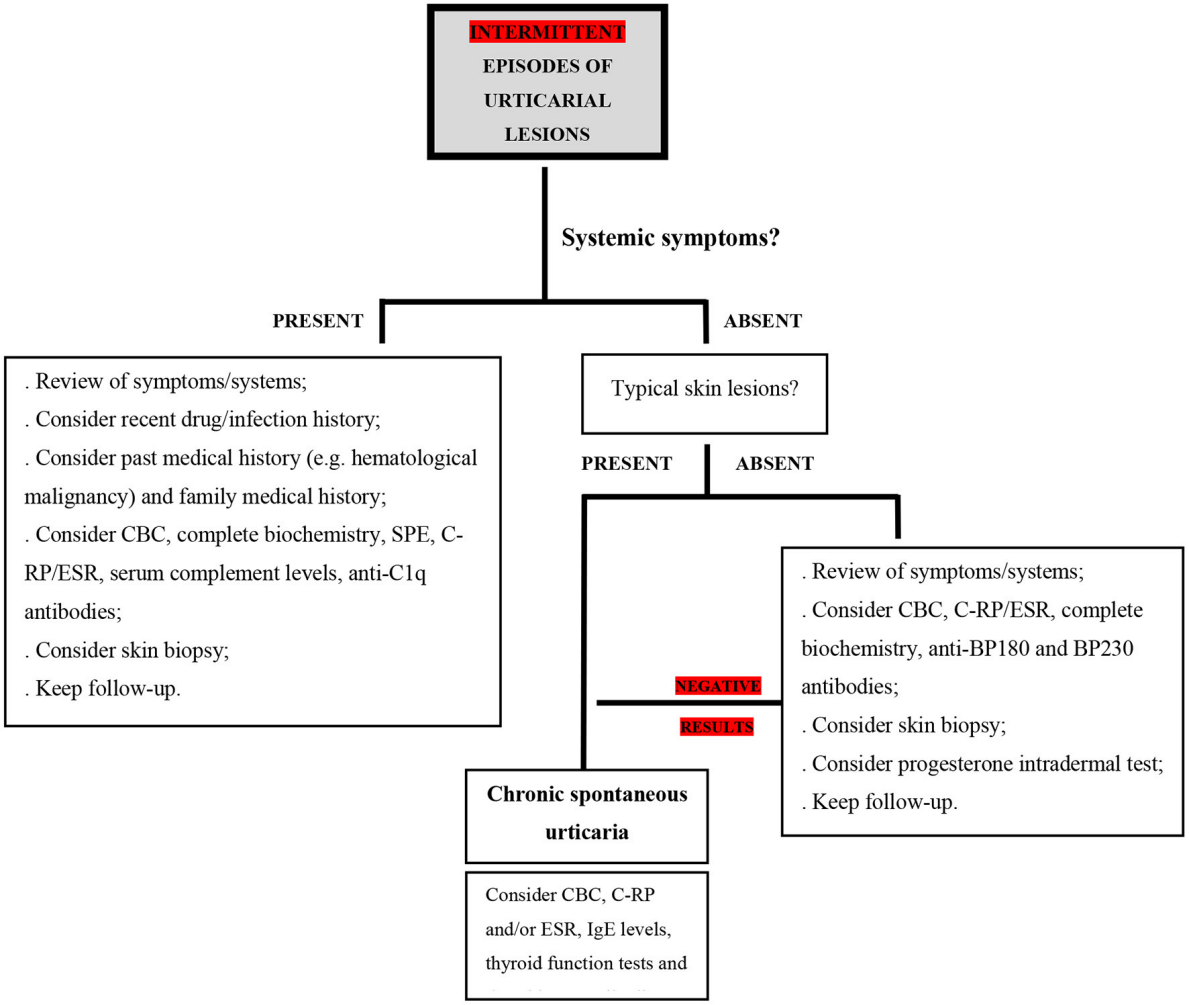
Proposed diagnostic algorithm in the setting of intermittent urticarial lesions; CBC, complete blood count; C-RP, C-reactive protein; ESR, erythrocyte sedimentation rate; SPE, serum protein electrophoresis; IgE, immunoglobulin E; BP180, BP 230 - bullous pemphigoid antigens BP230 and BP180.

If a patient reports intermittent crops of wheals for a period longer than 6 weeks, often with angioedema, the diagnosis of CU is likely. However, when accompanied by systemic symptoms or presenting with atypical characteristics, additional diagnoses must be ruled out. Systemic symptoms should alert to the possibility that an urticarial rash is not urticaria but rather a systemic syndrome with urticaria-like skin lesions (7). Also, these need to be suspected in patients who are refractory to standard CU treatment (Figure 2).

#### Urticarial Vasculitis

Urticarial vasculitis (UV) is characterized by recurrent urticarial lesions that remain fixed for more than 24 h and have histopathologic findings of leukocytoclastic vasculitis (17). Skin lesions slowly change in size and shape, can be painful, and often resolve with bruising or post-inflammatory hyperpigmentation (18). UV can also present with angioedema, purpura, extracutaneous manifestations related to systemic vasculitis such as arthralgia, lymphadenopathy, abdominal pain, ocular and renal manifestations or dyspnea/cough. It is usually idiopathic, but it can be associated with drugs, infections, malignancy or autoimmunity (17, 19). The diagnosis is ultimately based on cutaneous histopathology, but suggested laboratory studies include a complete blood count, serum creatinine, C-reactive protein (C-RP), erythrocyte sedimentation rate (ESR), urinalysis, complement studies (C1q, C3, C4), anti-C1q antibody assays and tests for underlying connective tissue disease or viral infection. The levels of complement divide UV into normocomplementemic (NUV), hypocomplementemic (HUV) or hypocomplementemic urticarial vasculitis syndrome (HUVS) (19). About 80% of all UV patients have NUV (19) which can be difficult to distinguish, on a clinical or even histopathological level, from severe forms of CSU (20, 21). HUV and HUVS are the most severe forms of UV and are often associated with longer disease duration and underlying disorders (17). Anti-C1q antibodies are found in about 55% of HUV patients, but they are not specific and may be observed both in patients with primary and secondary vasculitis (18).

#### Hypereosinophilic Syndromes

Hypereosinophilic syndromes constitute a heterogeneous group of disorders, characterized by a persistent and marked blood eosinophilia for more than 6 months, associated with evidence of eosinophil-induced organ damage, in the absence of other causes of hypereosinophilia, e.g., parasitosis. Cutaneous manifestations are common and nonspecific and generally consist of urticarial lesions, very itchy erythematous papules and nodules or eczematous lesions. Mucosal ulcerations are also possible (7). Cutaneous histopathology often shows dermal eosinophilic infiltration with typical flame figures.

#### Mast Cell Activation Syndrome

Mast cell activation syndrome (MCAS) is a recently described entity that may include primary (associated with clonality), secondary (a response to environmental triggers by normal mast cells) and idiopathic etiologies and can have cutaneous, gastrointestinal, cardiovascular, respiratory, and neurologic involvement. Cutaneous and subcutaneous manifestations include urticaria and angioedema that can be accompanied by anaphylaxis, flushing, nausea, vomiting, diarrhea, hypotension or tachycardia. MCAS remains a controversial diagnosis and has not been generally accepted. Some authors consider that the term MCAS should only be used in the idiopathic setting. However, some of the patients diagnosed with an idiopathic form are latter diagnosed with a clonal mast cell proliferative disease. Lastly, and even though some diagnostic criteria for MCAS have been proposed, this remains a complex topic and there are no definite diagnostic criteria identified (22).

#### Autoinflammatory Urticarial Syndromes

Autoinflammatory urticarial syndromes are rare and debilitating chronic diseases that can present with recurrent urticarial lesions with neutrophilic rich infiltrates on cutaneous histopathology, neutrophilic leukocytosis and elevated inflammation markers such as C-RP, ESR and serum amyloid A (SAA) (6, 18, 23). Lesions are usually flat erythematous wheals that last up to 24 h, are distributed mainly on the trunk and/or extremities and do not respond to H1-antihistamines. Pruritus may be absent, and lesions can be painful. These disorders are often diagnosed with a delay of several years (6) and may be hereditary or acquired. Cryopyrin-associated periodic syndromes are hereditary autoinflammatory diseases characterized by episodes of fever, urticaria-like rash, fatigue, headaches, arthralgia, arthritis, myalgia, sensorineural hearing loss, ocular inflammation, and/or bone lesions. They often manifest in early childhood. Inflammation is caused by an inappropriate activation of the innate immunity and overproduction of the proinflammatory cytokine interleukin-1 (18). Schnitzler syndrome is an acquired autoinflammatory disease that usually starts later in life and is characterized by recurrent fever, urticarial lesions, arthralgia, arthritis, myalgia, lymphadenopathy, hepatosplenomegaly and monoclonal gammopathy (mostly IgM class). About 15% of patients develop a lymphoproliferative disorder (23). Its pathophysiology remains unclear, but it is assumed to be IL-1 mediated (18, 23, 24). Anti-IL1 drugs can effectively control the disease but if left untreated, chronic inflammation may cause amyloidosis (18, 23). Adult-onset Still disease is a rare systemic inflammatory disease and usually manifests as a triad of high fever, arthralgia and an erythematous evanescent rash that accompanies the fever spike. Urticarial eruptions displaying neutrophilic infiltrates in histopathology occur in about 22% of the cases. IL-1 has also been implicated in its pathogenesis and, along with other acute inflammatory parameters, serum ferritin is usually significantly elevated. Neutrophilic urticarial dermatosis has also been reported as the presenting feature in systemic juvenile idiopathic arthritis, a closely related entity (25). Gleich syndrome (episodic angioedema with eosinophilia) is characterized by cyclic episodes of angioedema, wheals, fever, characteristic weight gain and dramatic eosinophilia (26).

If an autoinflammatory disease is suspected, testing for elevated inflammatory markers, serum protein electrophoresis to rule out monoclonal gammopathy in adults, urinalysis to screen for proteinuria due to secondary renal amyloidosis and skin biopsy to look for neutrophil-rich infiltrates are indicated. If a hereditary autoinflammatory disease is suspected, testing for mutations in the relevant genes should also be considered.

## Differential Diagnosis Of Angioedema Without Wheals

Angioedema without wheals represents a distinct clinical pattern and evokes several differential diagnoses (Table 2). Early diagnosis is essential since effective treatment depends on the main subtype and the main mediator responsible for increased vascular permeability (26, 27).

**Table 2:** Differential diagnosis in patients with angioedema.

**Subtype of angioedema**	**Mast cell-dependent angioedema**	**Bradykinin-mediated angioedema**			
		**Hereditary**		**Non-hereditary**	
		**Types I – II**	**Normal C1-INH**	**Drug-induced**	**Acquired C1-INH deficiency**
Associated urticaria	Frequent	No	No	No	No
Hereditary	No	Yes	Yes	No	No
Systemic symptoms	Not in CSU If acute, possible anaphylaxis	**Life-threatening oropharyngeal swellings** **Pseudo-occlusive abdominal crisis**			
Laboratory		Low C4, C1-INH	Genetic studies		Low C4, C1-INH
Culprits drugs/diseases	Possible NSAID, …	No	No	ACEi, ARA-II DPP-IVi sacubitril	Lymphoma Auto-immune diseases

*C1-INH, complement component 1 esterase inhibitor; CSU, chronic spontaneous urticaria; C4, complement component 4; ACEi, angiotensin-converting enzyme inhibitors; ARA-II, angiotensin II receptor antagonists; DPP-IVi, dipeptidyl peptidase 4 inhibitors; NSAID, non-steroidal anti-inflammatory drugs*.

### Mast Cell-Mediated Angioedema

Mast cell-mediated angioedema is triggered by histamine and other mast cell mediators. It responds well to H1-antihistamines, glucocorticoids and adrenaline. Around 10% of CSU patients have angioedema without wheals (28) and in this setting, angioedema can last up to 72 h (6) and commonly starts on the head or neck in the early morning hours (27). Mast cell-mediated angioedema may also occur in acute urticaria or during anaphylaxis. IgE-independent mechanisms of mast cell activation may also be involved in angioedema caused by drugs such as vancomycin or fluoroquinolones *via* Mas-related G protein-coupled receptor X2 (MRGPRX2) or non-steroidal anti-inflammatory drugs *via* alterations in arachidonic acid metabolism.

### Bradykinin-Mediated Angioedema

Bradykinin-mediated angioedema is triggered by bradykinin that promotes vasodilatation and increases vascular permeability. After phosphorylation of endothelial cadherins induced by bradykinin, adhesions between endothelial cells are opened, therefore causing plasma leakage with edema of the dermis and subcutis (angioedema), but no wheals. This type of angioedema responds poorly to standard CU medications, lasts up to 3–5 days and may cause a life-threatening swelling of the larynx and oropharynx and edema of gastrointestinal tract with occlusive symptoms, that often mimic a surgical abdominal emergency (27, 28).

Drugs, particularly angiotensin-converting enzyme inhibitors (ACEi) and less frequently angiotensin II receptor antagonists (ARA-II), dipeptidyl peptidase 4 (DPP-IV) inhibitors and sacubitril, involved in kinin degradation, have been associated with bradykinin-mediated angioedema (29). ACEi-associated angioedema is relatively common and may occur months, or even years, after onset of the drug. It usually resolves slowly after drug withdrawal, but some patients may have recurrent angioedema for months after ACEi withdrawal (30).

### Hereditary Angioedema

Hereditary angioedema can begin early in life or only after adolescence/early adulthood and is mainly due to autosomal dominant mutations in C1 inhibitor (C1-IHN) gene. Quantitative or functional C1-INH deficiency is associated with consumption of complement (low C4), but also uncontrolled activation of kallikrein and kininogen, which results in bradykinin overproduction. Angioedema attacks occur either spontaneously or triggered by minor stimuli like trauma or stress and may be life-threatening. Hereditary angioedema can occur with normal C1-INH, due to mutations in other genes involved in bradykinin overproduction, e.g. factor XII (Hageman Factor), plasminogen gene, angiopoeitin-1 gene and kininogen-1 gene, but there are still many unclassified cases of hereditary angioedema (31). Angioedema due to acquired C1-INH deficiency is often accompanied by a lymphoproliferative or autoimmune disorder that leads to continuous activation of the classic complement pathway with consequent depletion of C1-INH (32). Any patient with recurrent angioedema without wheals nonresponsive to standard CU treatment, not taking ACE inhibitors, should be screened for complement deficiency. If C4 level is low, C1-INH quantification and function need to be determined (27).

Angioedema also needs to be distinguished from other conditions characterized by swellings, especially when standard angioedema treatments fails. Granulomatous cheilitis is characterized by intermittent lip swelling at an initial stage, followed by persistent swelling of the lips, occasionally extending to the face due to granulomatous inflammation of unknown cause (26). In cellulitis and erysipelas there is acute inflammation of dermal and subcutaneous tissue due to a bacterial infection and the area involved becomes bright red, swollen, painful and hot usually with high fever and accompanying systemic symptoms. Wells syndrome (eosinophilic cellulitis) presents with a swelling resembling cellulitis (11). Autoimmune hypothyroidism, dermatomyositis and Sjögren's syndrome may present with periorbital swelling resembling angioedema of the eyelids (26). Allergic contact dermatitis, particularly related with hair dye allergy, may be misdiagnosed as facial angioedema. Initial clinical differentiation from angioedema may be challenging, but the swelling in contact dermatitis slowly spreads in the direction of gravity and clinical signs reflecting epidermal changes, like vesicles, scale and crusting, are present and regress faster if treated with glucocorticoids. Patch testing is required to confirm hypersensitivity to *p*-phenylenediamine and related chemicals used in hair dyes (30). Photoallergy, either from exposure to systemic drugs or from contact with photoallergens (non-steroidal anti-inflammatory drugs or sunscreens) usually appears several hours to days after exposure. It presents as a dermatitis, sometimes with important edema, and can be misdiagnosed as angioedema (33).

## Conclusion

A significant diagnostic challenge lies on the differentiation of common urticaria from urticarial syndromes or other dermatologic conditions that present with urticarial lesions and/or angioedema. Adding to the substantial value of a comprehensive clinical history and evaluation of skin lesions, skin biopsy, always supported by the clinician's perspective, may be of extreme value in these clinical settings. Looking for serum inflammatory parameters, like C-RP and ESR, leukocytosis, or other more clinically oriented biomarkers (C1q, C3, C4, ferritin, protein immunofixation, specific IgE, tryptase, ferritin) may also contribute to solve the puzzle of the differential diagnosis of urticarial lesions.

## Author Contributions

AM and MG contributed equally to manuscript writing. All authors contributed to manuscript revision, read, and approved the submitted version.

## Conflict of Interest

The authors declare that the research was conducted in the absence of any commercial or financial relationships that could be construed as a potential conflict of interest.

## Publisher's Note

All claims expressed in this article are solely those of the authors and do not necessarily represent those of their affiliated organizations, or those of the publisher, the editors and the reviewers. Any product that may be evaluated in this article, or claim that may be made by its manufacturer, is not guaranteed or endorsed by the publisher.
